# SGLT1/2 as the potential biomarkers of renal damage under Apoe^−/−^ and chronic stress *via* the BP neural network model and support vector machine

**DOI:** 10.3389/fcvm.2022.948909

**Published:** 2022-08-08

**Authors:** Gai-feng Hu, Xiang Wang, Ling-bing Meng, Jian-yi Li, Hong-xuan Xu, Di-shan Wu, Meng-jie Shan, Yu-hui Chen, Jia-pei Xu, Tao Gong, Zuoguan Chen, Yong-jun Li, De-ping Liu

**Affiliations:** ^1^Department of Cardiology, Beijing Hospital, National Center of Gerontology, Institute of Geriatric Medicine, Chinese Academy of Medical Sciences, Beijing, China; ^2^Graduate School, Chinese Academy of Medical Sciences and Peking Union Medical College, Beijing, China; ^3^Department of Plastic Surgery, Peking Union Medical College Hospital, Beijing, China; ^4^Department of Neurology, Beijing Hospital, National Center of Gerontology, Institute of Geriatric Medicine, Chinese Academy of Medical Sciences, Beijing, China; ^5^Department of Vascular Surgery, Beijing Hospital, National Center of Gerontology, Institute of Geriatric Medicine, Chinese Academy of Medical Sciences, Beijing, China; ^6^Department of Cardiology, The First Affiliated Hospital of Wenzhou Medical University, Zhejiang, China

**Keywords:** SGLT1, SGLT2, support vector machine, chronic stress, atherosclerosis, kidney

## Abstract

**Background:**

Chronic stress (CS) could produce negative emotions. The molecular mechanism of SGLT1 and SGLT2 in kidney injury caused by chronic stress combined with atherosclerosis remains unclear.

**Methods:**

In total, 60 C57BL/6J mice were randomly divided into four groups, namely, control (CON, *n* = 15), control diet + chronic stress (CON+CS, *n* = 15), high-fat diet + Apoe^−/−^ (HF + Apoe^−/−^, *n* = 15), and high-fat diet + Apoe^−/−^ + chronic stress (HF+Apoe^−/−^ + CS, *n* = 15) groups. The elevated plus maze and open field tests were performed to examine the effect of chronic stress. The expression of SGLT1 and SGLT2 in the kidney was detected. The support vector machine (SVM) and back propagation (BP) neural network model were constructed to explore the predictive value of the expression of SGLT1/2 on the renal pathological changes. The receiver operating characteristic (ROC) curve analysis was used.

**Results:**

A chronic stress model and atherosclerosis model were constructed successfully. Edema, broken reticular fiber, and increased glycogen in the kidney would be obvious in the HF + Apoe^−/−^ + CS group. Compared with the CON group, the expression of SGLT1/2 in the kidney was upregulated in the HF + Apoe^−/−^ + CS group (*P* < 0.05). There existed positive correlations among edema, glycogen, reticular fiber, expression of SGLT1/2 in the kidney. There were higher sensitivity and specificity of diagnosis of SGLT1/2 for edema, reticular fiber, and glycogen in the kidney. The result of the SVM and BP neural network model showed better predictive values of SGLT1 and SGLT2 for edema and glycogen in the kidney.

**Conclusion:**

In conclusion, SGLT1/2 might be potential biomarkers of renal damage under Apoe^−/−^ and chronic stress, which provided a potential research direction for future related explorations into this mechanism.

## Introduction

Atherosclerosis is progressive lipid accumulation in the major medium-stretch muscle-type artery intima. The formation of a fiber inflammatory lipid plaque (porcinoma) is a pathological feature of arterial disease. Pathological changes in the continuous development could cause luminal stenosis and a series of secondary change. The main manifestations include ischemic heart disease, myocardial infarction, and stroke. Atherosclerosis is the most common disease of the cardiovascular system (1). It occurs most frequently in middle-aged and elderly people. In China, the incidence and mortality of atherosclerosis are increasing significantly (2, 3).

Chronic stress is a physical and mental reaction of individuals facing long-term unpredictable stress, which will produce anxiety, depression, despair, and other negative emotions. At this time, the endocrine system of the body is disordered (4), which includes the sympathetic nervous system (SNS) and the hypothalamic–pituitary–adrenal axis (HPA). After the activation of the SNS and HPA axis, the release of catecholamine hormones, including epinephrine and norepinephrine, is an important stress response (5, 6), which act on the adrenergic α and β receptors. Chronic stress is not only related to the occurrence and aggravation of a variety of adverse psychological states but also has a significant interference effect on the level of inflammatory factors and the central system of the body, which will lead to occurrence of many diseases. However, the molecular mechanism of kidney injury caused by chronic stress combined with atherosclerosis remains unclear, which may be related to genetic factors, chromosomal abnormalities, gene fusion, and other factors. Therefore, it is particularly important to study the molecular mechanism of chronic stress combined with atherosclerosis on kidney injury.

Sodium–glucose cotransporters (SGLTs) are a family of glucose transporters found in the small intestinal mucosa and the proximal convoluted tubules of the kidney. Their function is to mediate the transmembrane transport of glucose in the kidney and intestinal tract (7). It was confirmed that this heterogeneity was due to two different transporters, namely, SGLT1 and SGLT2 (8, 9). There are 12 members of sodium–glucose cotransporters; six of those are named SGLTSs, among which SGLTI and SGLT2 transporters have been studied increasingly. SGLT1 is located at q13.1 on chromosome 22, and SGLT2 is located at p12-pl1 on chromosome 16. Overall, 59% of the amino acids of the two transporters encoded are homologous (8, 10). When SGLT expression is abnormal, it may lead to kidney injury, but its relationship with chronic stress combined with atherosclerosis in kidney injury remains unclear.

Therefore, this study intends to construct an animal model of atherosclerosis and chronic stress, observe the status of kidney injury initially, and further explore the differences in the expression of SGLT1 and SGLT2 in the kidney so as to explore the potential impact of their combined effects on kidney injury under the condition of chronic stress combined with atherosclerosis.

## Methods

### Animal and diets

In total, 60 C57BL/6J mice (male, 18–20 weeks old, 27.10 ± 0.43 g) were obtained from Huafukang Biotechnology Co., Ltd. (Beijing, China), which were randomly divided into four groups, namely, control (CON, *n* = 15), control diet + chronic stress (CON + CS, *n* = 15), high-fat diet + Apoe^−/−^ (HF + Apoe^−/−^, *n* = 15), and high-fat diet + Apoe^−/−^ + chronic stress (HF + Apoe^−/−^+CS, *n* = 15) groups. All animals were fed under appropriate laboratory conditions, that is, at a temperature range of 20–25°C and a relative humidity of 40–70%. In addition, the light/dark cycle was 12/12 h (7:00 am−7:00 pm), except for chronic stress model mice (CON + CS, HF + Apoe^−/−^ + CS), which were kept in the experimental environment. The Animal Care and Use Committee of the Institute of Laboratory Animal Sciences, Chinese Academy of Medical Sciences and Peking Union Medical College (CAMS and PUMC), authorized the experimental ethics agreement.

The mice in the CON and CON + CS groups were fed with the maintenance fodder (1,022, Huafukang Biotechnology Co., Ltd., Beijing, China), and the mice in the other two groups were fed with Western diets (fat content = 21%, high-fat diet, H10141, Huafukang Biotechnology Co., Ltd., Beijing, China).

### Construction of the chronic stress model

The mice in the CON + CS and HF + Apoe^−/−^ + CS were applied to chronic stress for 12 weeks. A proper combination of stressors could avoid the adaptation of mice to the stress regimen. To avoid habituation, the researcher randomly performed the following stress routines each week (Table 1): (1) Clamp tail: clamp 1 cm from the end of rat tail. (2) Sudden vibration: shake the cage for 5 s each time at an interval of 10 s. (3) Day/night reversal: reverse the light cycle (animals exposed to continuous indoor light for 24 h) into the reverse photoperiod mode, followed by 48 h of reverse 12-h on/off photoperiod, and then holding light for 24 h to re-enter the normal 12-h on/off photoperiod. (4) Predation stress: The experimental mice were placed in a cage with rats and exposed to rat odor. (5) Noise stimulation: Exposure to white noise at 70 dB. (6) Flash stimulation: low-intensity strobe lighting (150 flashes/min). (7) Bind: bind the mouse in a fixator, unable to turn around. (8) Squirrel cage tilt: The squirrel cage is placed on a platform with a tilt angle of 45 degrees, and the tilt angle is rotated 180 degrees every hour. (9) Wet dressings.

**Table 1:** Procedure of chronic stress for 12 weeks.

**Time**	**Monday**	**Tuesday**	**Wednesday**	**Thursday**	**Friday**	**Saturday**	**Sunday**
Week 1 7 weeks	Baseline data	Damp pad for 24 h Clip tail, 1 min	Flash stimulation for 7 h The cage tilts for 7 h	The noise stimulated for 9 h Predation stress 1 h	Constraints in 2 h Oscillate and clip tail for 1 min		
2 weeks 8 weeks	Constraints in 2 h	Day and night upside down The noise was stimulated for 15 h	The cage tilts for 7 h Oscillate and clip tail for 1 min	Damp pad for 24 h Flash stimulation for 12 h	Flash stimulation for 12 h The noise stimulated for 9 h		Constraint 4 h
3 weeks 9 weeks	Flash stimulation for 7 h	Predation stress 1 h Oscillating, clip tail 1 inm	Constraints in 2 h	The noise stimulated for 9 h The clip tail 1 min	Damp pad for 24 h	The noise was stimulated for 15 h Constraint 4 h	Flash stimulation for 7 h The clip tail 1 min
4 weeks 10 weeks	Predation stress 1 h Constraint 4 h	The noise was stimulated for 15 h The clip tail 1 min	Day and night upside down Oscillate and clip tail for 1 min	Damp pad for 24 h	The noise stimulated for 9 h		Constraints in 2 h Flash stimulation for 7 h
5 weeks 11 weeks	The noise was stimulated for 15 h	Flash stimulation for 12 h	Damp pad for 24 h	Constraints in 2 h Day and night upside down	Predation stress 1 h	The noise stimulated for 9 h Oscillate and clip tail for 1 min	Constraint 4 h The flash stimulated for 9 h
6 weeks Week 12	Day and night upside down The noise stimulated for 9 h	Damp pad for 24 h The flash stimulated for 9 h	The cage tilts for 7 h Oscillate and clip tail for 1 min	Constraints in 2 h	The noise was stimulated for 15 h The clip tail 1 min	Predation stress 1 h The clip tail 1 min	

### Elevated plus maze assay to examine the effect of chronic stress

The elevated plus maze was purchased from Nanjing Calvin Biotechnology Co., LTD. (Nanjing, China). In order to increase the total number of mice entering the arm and avoid rats hiding in the closed arm, mice are usually placed in the open field for 5 min before being put into the maze. Petting animals for 1–2 min a day for 5–7 days before the experiment also reduced the influence of unrelated stress stimuli on the experiment. The mice were placed in the center of the maze, with the observer at least 1 m away from the center. The average velocity of each mouse was recorded during the experimental period (usually 5 min) in total field, open arms, closed arms, and central region, which was defined as the indicator of evaluating anxiety.

### Open field test to examine the effect of chronic stress

The open field test is a method to evaluate the autonomous behavior, exploratory behavior, and tension of experimental animals in a new environment. The frequency and duration of some behaviors in the novel environment were used to reflect the autonomous behavior and exploratory behavior of experimental animals in the unfamiliar environment. The size of the open field device is 50 cm × 50 cm × 40 cm, which was obtained from Nanjing Calvin Biotechnology Co., LTD. (Nanjing, China). The mice were placed in the central area of the open field experiment reaction box. The bottom part of the reaction box had 16 cells, the peripheral 12 cells were recorded as the peripheral area, and the middle four cells were recorded as the central area. The camera and timing were performed at the same time. After a certain period of observation, the camera was stopped. The observation time is generally 3–5 min. The locomotive speed of mice in different areas within 5 min was recorded. The open field test was performed in a soundproof chamber without any human interference. After each mouse was tested, the reaction chamber was swabbed with 5% water–ethanol solution to eliminate the bias caused by the odor of the previous mice.

### Obtaining tissues

After the experiment of establishing the animal model, the animals were narcotized with 4.5% isoflurane (0.8–1L/min), and the perfusion of heart was performed. The death of the mice was judged by cardiac and breathing arrest.

The whole abdominal aorta was removed and embedded in Tissue-Tek OCT compound (Sakura, Tokyo, Japan) for frozen sectioning. The kidney was removed and would be embedded in Tissue-Tek OCT compound (Sakura, Tokyo, Japan) for frozen sectioning, hematoxylin–eosin (HE) staining, immunohistochemistry, and immunofluorescence assays.

### Hematoxylin–eosin staining

The embedding agent was removed, and undecalcified bone sections were rehydrated using ethylene glycol ethyl ether acetate (Macklin, E808814-2.5L) and alcohol. The sections were stained with hematoxylin solution and left for 8–10 min. Then rinse the section with tap water. Treat the section with Hematoxylin Scott Tap Bluing, rinse with tap water. The sections were dehydrated with 85% ethanol for 5 min; 95% ethanol for 5 min; Finally Stain sections with Eosin dye for 5 min. Finally, absolute ethanol was used to dehydrate; xylene to transparency, neutral balsam to mount sections. Then images were obtained under an optical microscope (Nikon Eclipse E100); the microscopic analysis revealed that the nucleus was blue and the cytoplasm was red.

### Immunofluorescence

After washing three times with PBS (pH 7.4) (5 min/time), the renal sections were immersed in EDTA antigen retrieval buffer (pH 8.0) (Servicebio G1206, Wuhan, China) for antigen retrieval. After that, the sections were treated with PBS (pH 7.4) (three times, 5 min/time), and then 3% BSA (Servicebio, G5001, Wuhan, China) was added and left for 30 min to block non-specific binding. After discarding the blocking solution, the sections are incubated with the SGLT1 antibody (dilution rate = 1:500, bs-1128R, BIOSS, Beijing, China) (overnight, at 4°C). Then the sections were again washed with PBS (pH 7.4), and fluorescent secondary antibodies (dilution rate = 1:5000) responding to the primary antibodies were added (room temperature, 50 min, dark condition). Then, the solutions were incubated with DAPI solution (Servicebio, G1012, Wuhan, China) (room temperature, 10 min, darkness) for counterstaining the nucleus.

Finally, spontaneous fluorescence quenching reagent (5 min) (Servicebio, G1221, Wuhan, China) was used for spontaneous fluorescence quenching, and the sections were sealed with anti-fade mounting medium. The detection process of SGLT2 was the same as mentioned before using SGLT2 antibody (dilution rate = 1:200, 24654-1-AP, Proteintech, Rosemont, USA). Fluorescence microscopy (Nikon ECLIPSE C1) showed that the nuclei were blue (excitation wavelengths 330–380 nm and emission wavelength 420 nm), and the positive expression was red or green (FITC glows green at excitation wavelengths 465–495 nm and emission wavelengths 515–555 nm; CY3 glows red by excitation wavelengths 510–560 nm and emission wavelength 590 nm).

### Immunohistochemical assay

The renal samples were fixed with 4% paraformaldehyde. Citric acid (pH 9.0) was used as the antigen retrieval buffer (Servicebio G1203, Wuhan, China) for antigen retrieval. The sections were then incubated with 3% hydrogen peroxide (room temperature, darkness) for 25 min to block endogenous peroxidase. Then 3% BSA was added and left for 30 min at room temperature for blocking non-specific binding. After removing the blocking solution, the SGLT1 antibody (dilution rate = 1:500, bs-1128R, BIOSS, Beijing, China) was added to the sections and was incubated overnight at 4°C. The sections were then washed with PBS (pH 7.4) (three times, 5 min/time), slightly shaken, and dried. Following that, the sections were incubated with the secondary antibody (HRP-labeled) (dilution rate = 1:5000) at room temperature for 50 min. Freshly prepared DAB color developing solution was used for the DAB chromogenic reaction. The color developing time is controlled under a microscope. The sections were then rinsed with tap water to stop the reaction. The sections are counterstained with hematoxylin stain solution for about 3 minutes; differentiated with hematoxylin differentiation solution for several seconds; treated with hematoxylin returning blue solution. Different degrees of alcohol for dehydration, xylene for transparency, and neutral gum for mounting the sections were used. The process of detecting SGLT2 was the same as mentioned previously using the SGLT2 antibody (dilution rate = 1:200, 24654-1-AP, Proteintech, Rosemont, USA).

The nucleus was blue, and the positive expression of DAB is brownish yellow. The images were collected and analyzed under an optical microscope (Nikon Eclipse E100). ImageJ (National Institutes of Health, USA), a Java-based application for analyzing images, was applied to analyze the quantitative expression of the SGLT1 and SGLT2 in the kidney.

### Support vector machine

The support vector machine (SVM) is a kind of generalized linear classifier that classifies binary data by supervised learning. Its decision boundary is the maximum margin hyperplane solved for learning samples. The two input variables included the expression of SGLT1 and SGLT2. The output variable was renal edema, or broken reticular fiber, or glycogen content.

In order to improve the generalization ability of the network, the study adopted the regularization adjustment method to train the network. The regularization adjustment method enhances the generalization ability of the network by adjusting the performance function of the network.

### Construction of the BP neural network model

The topological structure of the BP neural network model includes the input layer, hidden layer, and output layer. For a given pair of sample patterns, a set of free weights is randomly selected as the fixed weights between the output layer and the hidden layer, and the actual output of the hidden layer is calculated by the transfer function. Then the weights between the output layer and the hidden layer are taken as the quantity to be solved, and the target output is directly taken as the right side of the equation to establish a system of equations to solve the problem. MATLAB (version 2014a) was used to accomplish the normalization process of variable values, network simulation, network training, and network initialization. The two input variables included the expression of SGLT1 and SGLT2. The output variable was renal edema, or broken reticular fiber, or glycogen content. The cubic spline interpolation algorithm was implemented to analyze the high-risk warning range of renal edema, broken reticular fibers, and the glycogen content with the expression of SGLT1 and SGLT2.

### Statistics

The results are presented as the mean ± standard error of the mean. When two groups were compared, an unpaired Student's *t*-test was performed to determine statistical significance. The Spearman and Pearson-rho tests were executed to compare SGLT1, SGLT2, renal edema, broken reticular fibers, and the glycogen content for the correlation analysis. The receiver operating characteristic (ROC) curve analysis was used to determine the sensitivity and specificity of SGLT1 and SGLT2 for predicting renal edema, broken reticular fibers, and the glycogen content. The statistical analyses were conducted using SPSS software, version 24.0 (IBM Corp., Armonk, NY, USA). A *p*-value < 0.05 was considered statistically significant.

## Results

### Extremely excited state in the chronic stress model based on the elevated plus maze test

**Figure 1:**
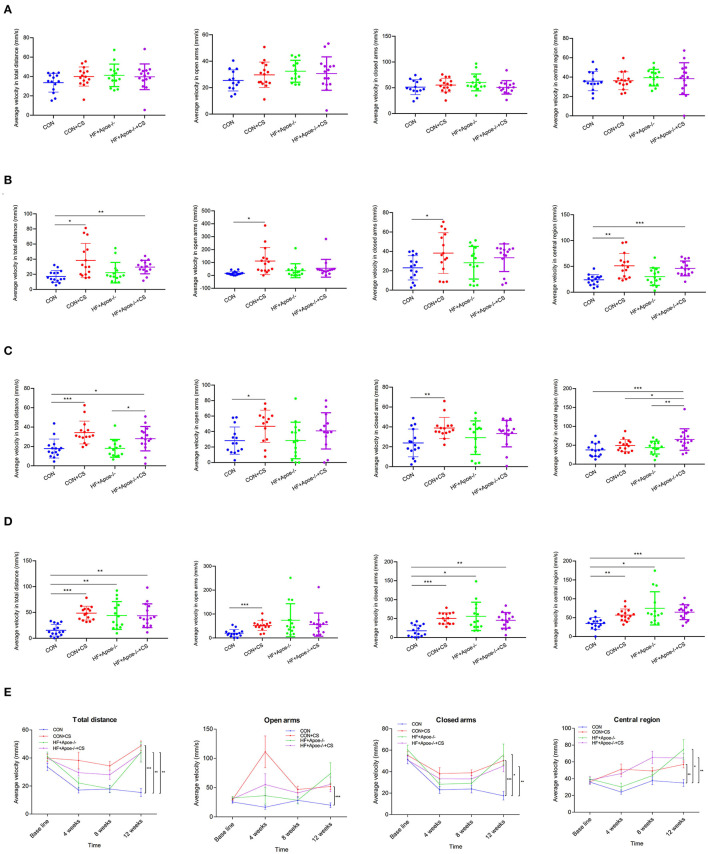
Extremely excited state in the chronic stress model based on the elevated plus maze. **(A)** Baseline. **(B)** Four weeks. **(C)** Eight weeks. **(D)** Twelve weeks. **(E)** Trend of excitation over time is shown in the line chart. *P < 0.05; **P < 0.01; ***P < 0.001.

After the elevated plus maze test, there was no difference in the baseline among the four groups (CON, CON + CS, HF + Apoe^−/−^, and HF + Apoe^−/−^+ CS) in the aspects of average velocity in total distance, open arms, closed arms, and central region, which could exclude individual differences (Figure 1A). After 4 weeks of chronic stress, average velocity in the total distance in the CON + CS (*P* < 0.05) and HF + Apoe^−/−^+ CS (*P* < 0.01) groups was higher than that of the CON group. The average velocity in open arms and closed arms in the CON + CS group were significantly increased compared to that in the CON group (*P* < 0.05). The average velocity in the central region in the Con + CS (*P* < 0.01) and HF + Apoe^−/−^+ CS (*P* < 0.001) groups was higher than that in the CON group (Figure 1B). After 8 weeks of chronic stress, the average velocity in total distance in the CON + CS (*P* < 0.001) and HF + Apoe^−/−^+ CS (*P* < 0.05) groups was higher than that in the CON group. Compared with the HF + Apoe^−/−^ group, the average velocity in total distance in the HF + Apoe^−/−^+ CS group was obviously increased (*P* < 0.05). The average velocity in open arms and closed arms in the CON + CS group was significantly increased compared to that in the CON group (*P* < 0.05). The average velocity in the central region in the HF + Apoe^−/−^+ CS group was significantly higher than that in the CON (*P* < 0.001), CON + CS (*P* < 0.05), and HF + Apoe^−/−^ (*P* < 0.01) groups (Figure 1C). After 12 weeks of chronic stress, compared with the CON group, the average velocity in total distance was significantly elevated in the CON + CS (*P* < 0.001), HF + Apoe^−/−^ (*P* < 0.01), and HF + Apoe^−/−^+CS (*P* < 0.01) groups. Compared with the CON group, the average velocity in open arms was higher in the CON + CS group (*P* < 0.001). Compared with the CON group, the average velocity in closed arms was increased in the CON + CS (*P* < 0.001), HF + Apoe^−/−^ (*P* < 0.05), and HF + Apoe^−/−^+CS (*P* < 0.01) groups. Compared with the CON group, the average velocity in the central region was increased in the CON + CS (*P* < 0.01), HF + Apoe^−/−^ (*P* < 0.05), and HF + Apoe^−/−^+CS (*P* < 0.001) groups (Figure 1D). In addition, the trend of excitation over time is shown in the line chart, which manifests the extremely excited state in the chronic stress model (Figure 1E).

### Verification of the successful construction of the chronic stress model *via* the open field test

**Figure 2:**
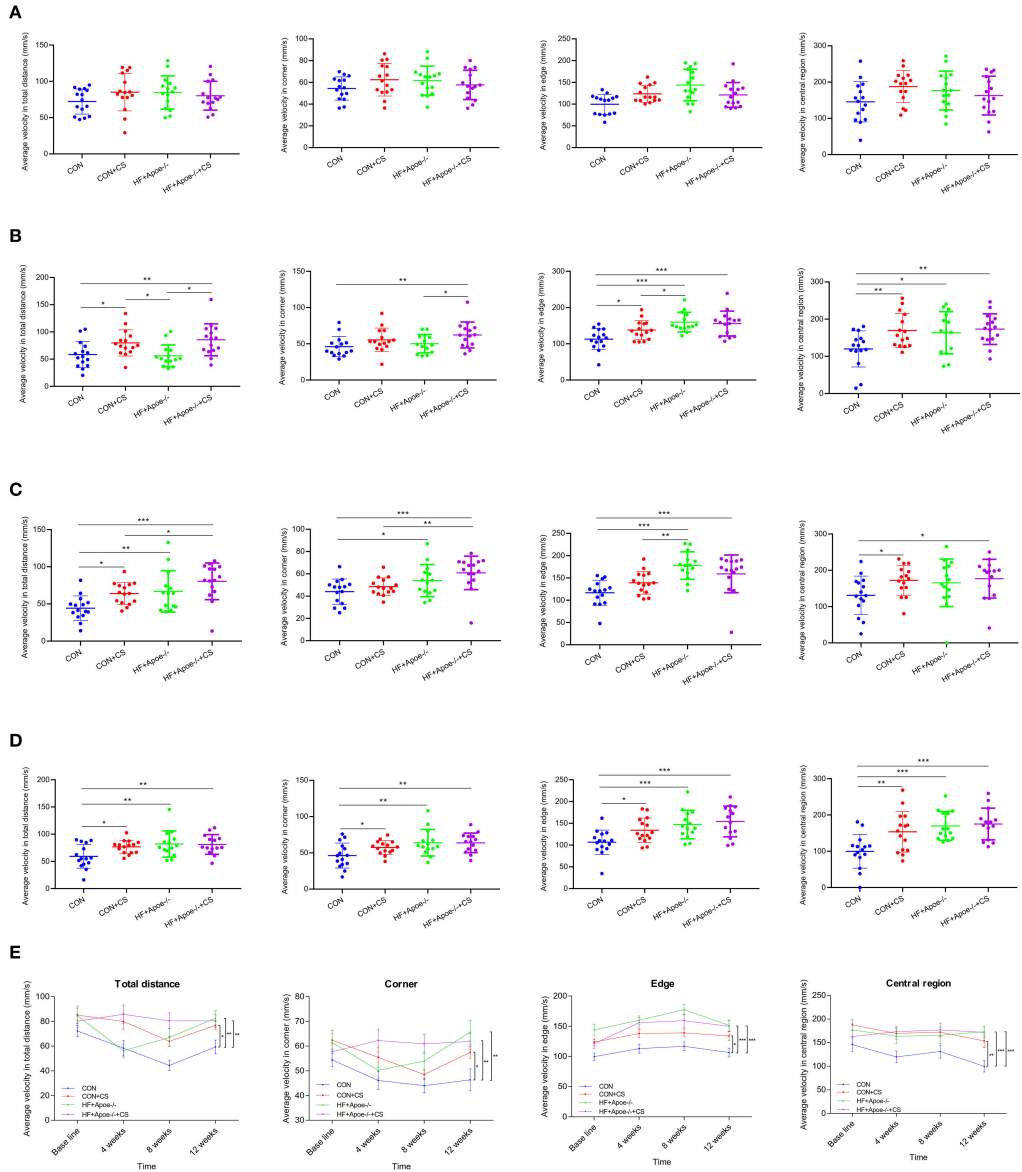
Verification of the successful construction of a chronic stress model *via* the open field test. **(A)** Baseline. **(B)** Four weeks. **(C)** Eight weeks. **(D)** Twelve weeks. **(E)** Trend of excitation over time is shown in the line chart. *P < 0.05; **P < 0.01; ***P < 0.001.

After the open field test, there was no difference in the baseline among the four groups (CON, CON + CS, HF + Apoe^−/−^, and HF + Apoe^−/−^+CS) in the aspects of average velocity in total distance, corner, edge, and central region, which could exclude individual differences (Figure 2A). After 4 weeks of chronic stress, the average velocity in total distance in the CON + CS (*P* < 0.05) and HF + Apoe^−/−^+CS (*P* < 0.01) groups was higher than that in the CON group. In addition, the average velocity in total distance was increased significantly in the HF + Apoe^−/−^+CS group than in the HF + Apoe^−/−^ group (*P* < 0.05). The average velocity in the corner in the HF + Apoe^−/−^+CS group was significantly increased compared to that the CON (*P* < 0.01) and HF + Apoe^−/−^ (*P* < 0.05) groups. Compared with the CON group, the average velocity in the edge was higher in the CON + CS (*P* < 0.05), HF + Apoe^−/−^ (*P* < 0.001), and HF + Apoe^−/−^+CS (*P* < 0.001) groups. The average velocity in central region in the CON + CS (*P* < 0.01), HF + Apoe^−/−^ (*P* < 0.05), and HF + Apoe^−/−^+CS (*P* < 0.01) groups was higher than that in the CON group (Figure 2B). After 8 weeks of chronic stress, compared with the CON group, the average velocity in total distance was increased in the CON + CS group (*P* < 0.05), HF + Apoe^−/−^ (*P* < 0.01), and HF + Apoe^−/−^+CS (*P* < 0.001) groups. The average velocities in the corner (*P* < 0.001), edge (*P* < 0.001), and central region (*P* < 0.05) in the HF + Apoe^−/−^+CS group were all revised compared to those in the CON group. Compared with the CON group, the average velocity in the central region in the CON + CS was higher (*P* < 0.05) (Figure 2C). After 12 weeks of chronic stress, compared with the CON group, the average velocity in total distance in the corner, edge, and central region was significantly elevated in the CON + CS, HF + Apoe^−/−^, and HF + Apoe^−/−^+CS (*P* < 0.05) groups (Figure 2D). In addition, the trend of average velocity in the total distance in the corner, edge, and central region over time was shown in the line chart, which manifests extremely excited state in the chronic stress model (Figure 2E).

### Construction of the atherosclerosis model

**Figure 3:**
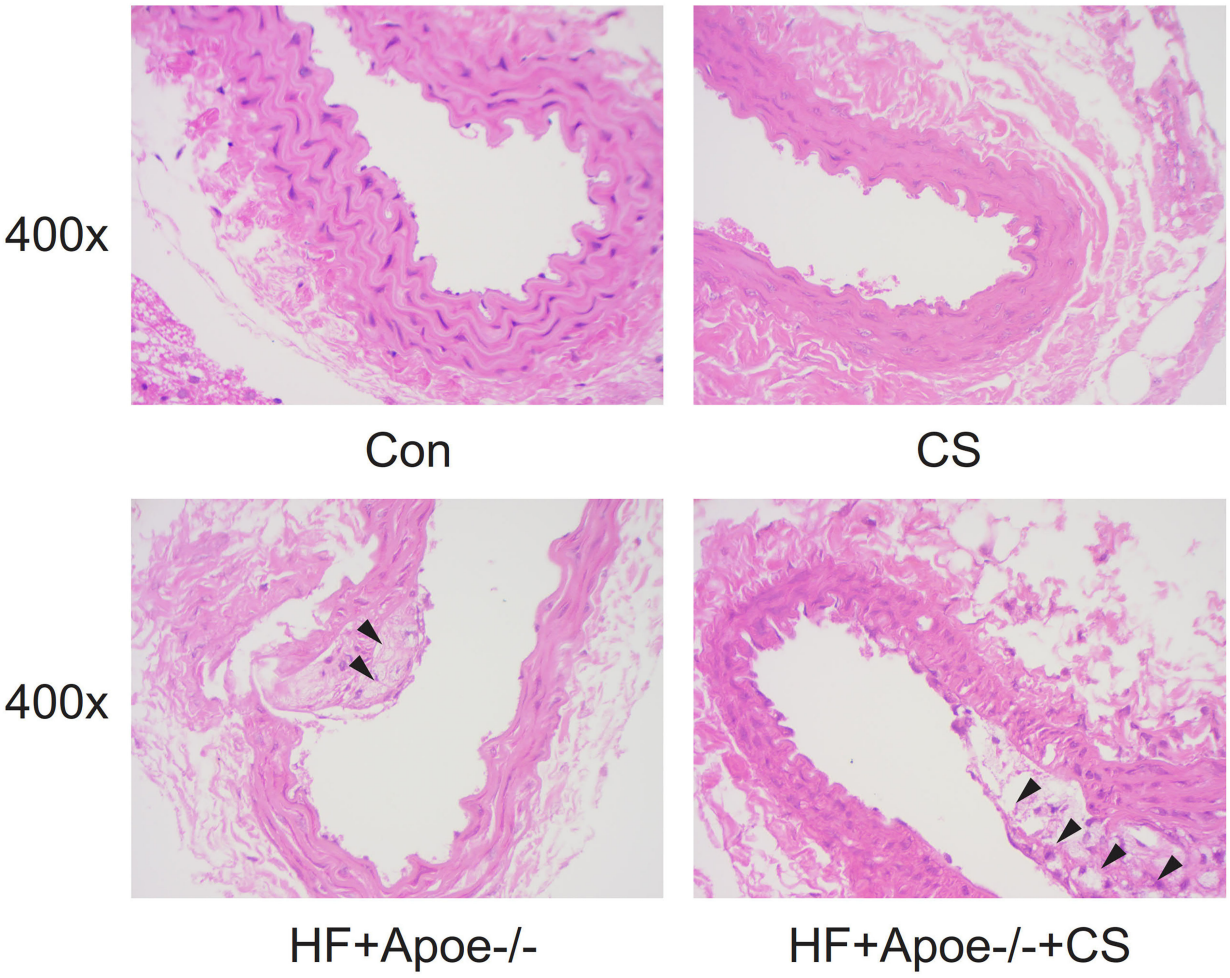
Pathological observation of the normal artery and atherosclerosis based on HE staining.

In the CON and CS groups, the arterial wall was thick and rich in elastic fibers, the lumen section was round, and the intima was smooth. However, in the HF + Apoe^−/−^ and HF + Apoe^−/−^+CS groups, there were deposition of lipid and other blood components, proliferation of smooth muscle cells, and increase in collagen fibers in the intima of the arteries, resulting in the formation of lipid-containing necrosis lesions and hardening of the vascular wall (Figure 3).

### Edema, broken reticular fiber, and increased glycogen in the kidney under the situation of HF + Apoe^–/-^+CS

**Figure 4:**
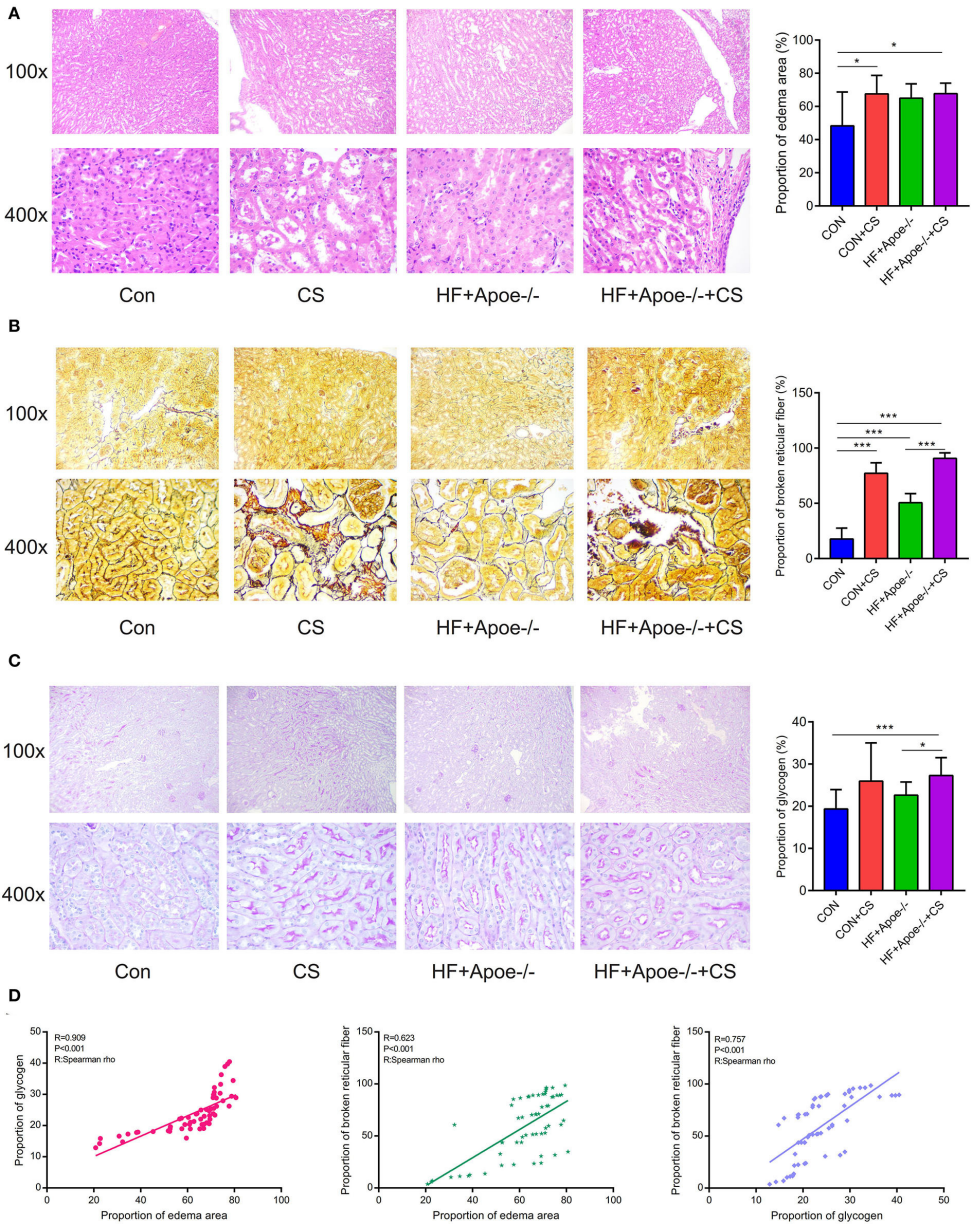
Edema, broken reticular fibers, and increased glycogen in the kidney under in the HF + Apoe^−/−^+CS group. **(A)** HE staining manifested that there existed edema in the kidney at different degrees in the CS, HF + Apoe^−/−^, and HF + Apoe^−/−^+CS groups. **(B)** In the CS, HF + Apoe^−/−^, and HF + Apoe^−/−^+CS groups, the reticular fibers in the kidney were destructed and broken. **(C)** Compared with the CON group, the proportion of glycogen in the HF + Apoe^−/−^+CS group was significantly higher than that in the CON group. **(D)** Strongly positive relationships between the proportion of glycogen, broken reticular fiber, and edema area in the kidney. *P < 0.05; ***P < 0.001.

HE staining manifested that there existed edema in the kidney to different degrees under the situation of CS, HF + Apoe^−/−^, and HF + Apoe^−/−^+CS. Compared with the CON group, the proportion of the edema area in the CON + CS and HF + Apoe^−/−^+CS groups was significantly increased compared to that in the CON group (*P* < 0.05), which presented that the CS might accelerate edema in the kidney (Figure 4A). After staining, the morphology and distribution of reticular fibers—which were stained black—were examined under a microscope. In CS, HF + Apoe^−/−^, and HF + Apoe^−/−^+CS groups, the reticular fibers in the kidney were destructed and broken. Compared with the CON group, the proportion of broken reticular fibers was higher in the CON + CS (*P* < 0.001), HF + Apoe^−/−^ (*P* < 0.001), and HF + Apoe^−/−^+CS (*P* < 0.001) groups. In addition, the proportion of broken reticular fibers in the HF + Apoe^−/−^+CS group was significantly increased compared to that in the HF + Apoe^−/−^ group (*P* < 0.001) (Figure 4B). In the periodic acid–Schiff staining, glycogen could be stained purplish red. In the CS, HF + Apoe^−/−^, and HF + Apoe^−/−^+CS groups, the level of glycogen in the kidney was revised in different degrees. Compared with the CON group, the proportion of glycogen in the HF + Apoe^−/−^+CS group was significantly higher than that in the CON (*P* < 0.001) and HF + Apoe^−/−^ (*P* < 0.05) groups (Figure 4C). The Spearman correlation analysis manifested that there was a strongly positive relationship between the proportion of glycogen and edema in the kidney (*R* = 0.909, *P* < 0.001). Also, the proportion of broken reticular fibers was significantly related with the edema area (*R* = 0.623, *P* < 0.001) and the proportion of glycogen (*R* = 0.757, *P* < 0.001) (Figure 4D).

### Upregulated expression of SGLT1 and SGLT2 in the kidney in the HF + Apoe^–/-^+CS group

**Figure 5:**
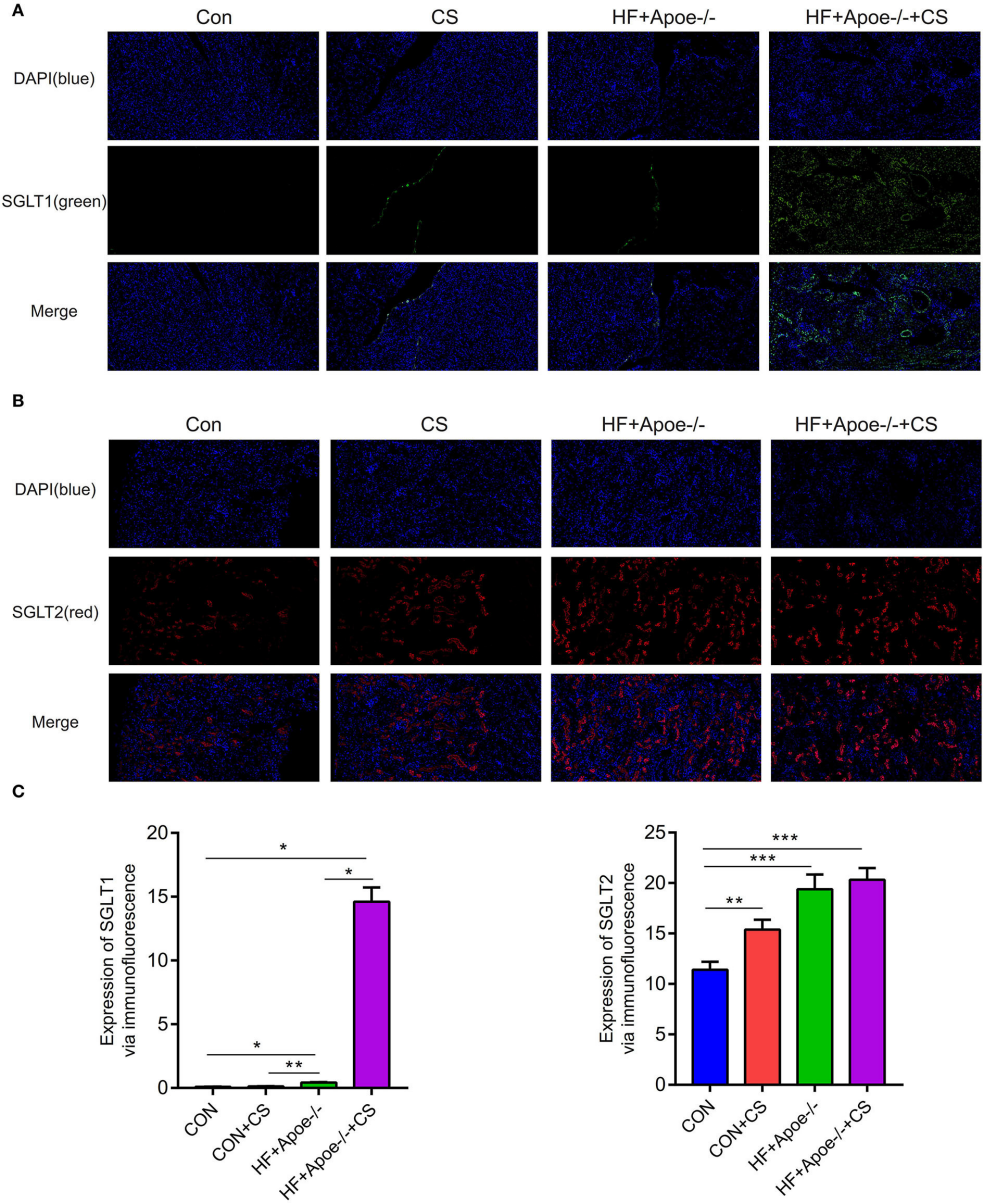
Upregulated expression of SGLT1 and SGLT2 in the kidney in the HF + Apoe^−/−^+CS group. **(A)** In the immunofluorescence assay, SGLT1 stained green. Compared with the CON group, the expression of SGLT1 in the kidney was upregulated in the HF + Apoe^−/−^ and HF+Apoe^−/−^+CS groups (*P* < 0.05). In addition, the expression of SGLT1 in the HF + Apoe^−/−^+CS group was higher than that in the HF + Apoe^−/−^ group. **(B)** In the immunofluorescence assay, SGLT2 stained red in the kidney. Compared with the CON group, the expression of SGLT2 in the kidney was upregulated in the HF + Apoe^−/−^ and HF + Apoe^−/−^+CS groups, and the expression of SGLT2 in the kidney was higher in the CON + CS group than in the CON group. **(C)** Statistical graph of the expression of SGLT1 and SGLT2 in the kidney. *P < 0.05; **P < 0.01; ***P < 0.001.

In the immunofluorescence assay, SGLT1 stained green and SGLT2 stained red in the kidney. Compared with the CON group, the expression of SGLT1 in the kidney was upregulated in the HF + Apoe^−/−^ and HF + Apoe^−/−^+CS groups (*P* < 0.05). In addition, the expression of SGLT1 in the HF + Apoe^−/−^+CS group was higher than that in the HF + Apoe^−/−^ group. Furthermore, compared with the CON group, the expression of SGLT2 in the kidney was upregulated in the HF + Apoe^−/−^ and HF + Apoe^−/−^+CS groups (*P* < 0.001), and the expression of SGLT2 in the kidney was higher in the CON+CS group than that in the CON group (Figure 5).

### Verification of expression of SGLT1 and SGLT2 in the kidney *via* the immunohistochemical assay

**Figure 6:**
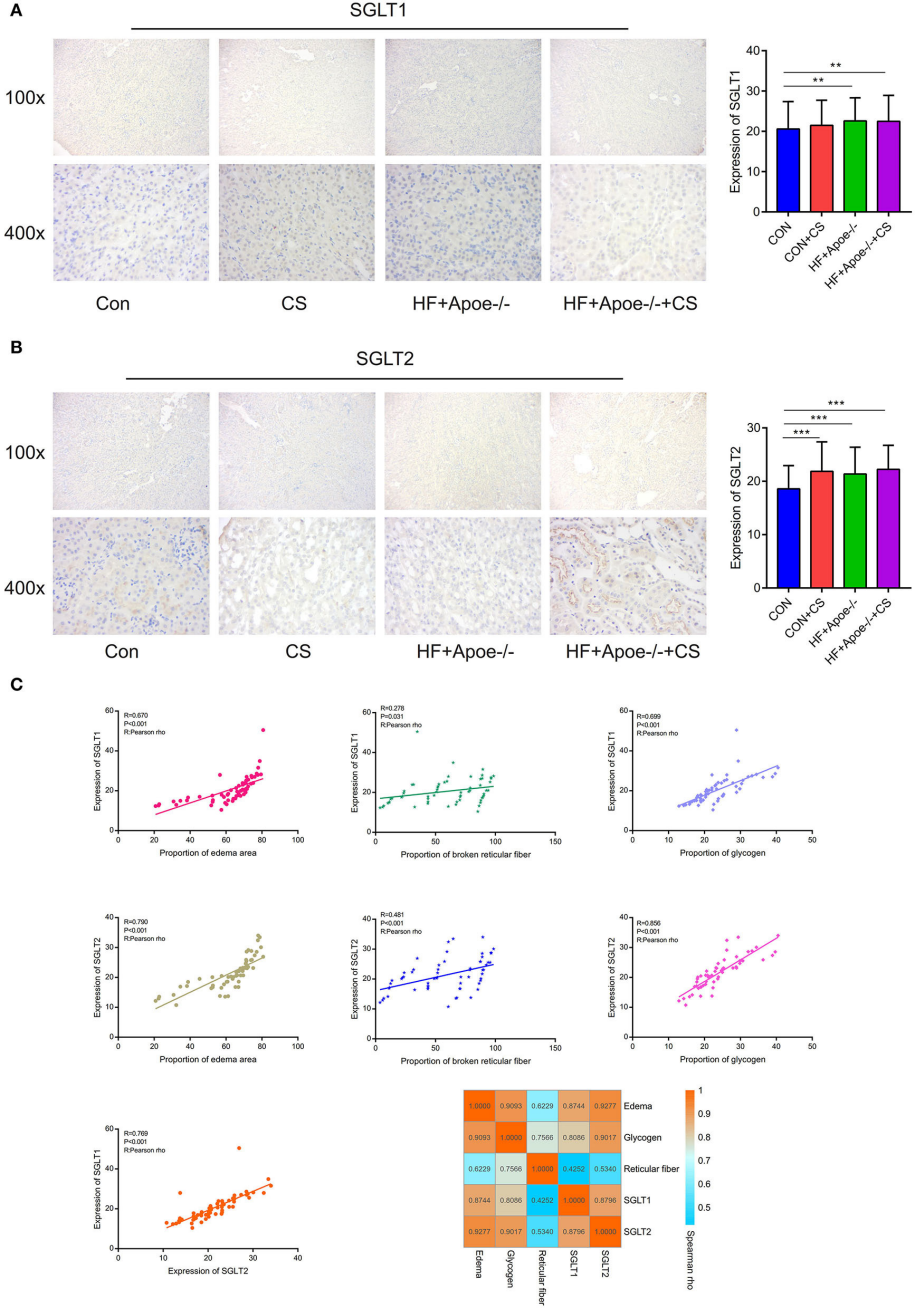
Verification of the expression of SGLT1 and SGLT2 in the kidney *via* the immunohistochemical assay. **(A)** Through the immunohistochemical assay, the expression of SGLT1 in the kidney in the HF + Apoe^−/−^ group and HF + Apoe^−/−^+CS group was upregulated compared with the CON group. **(B)** Compared with the CON group, the expression level of SGLT2 in the kidney was higher in the CON + CS, HF + Apoe^−/−^, and HF + Apoe^−/−^+CS groups. **(C)** Positive correlations edema, glycogen, reticular fiber, and expression of SGLT1 and SGLT2 in the kidney. **P < 0.01; ***P < 0.001.

In the immunohistochemical assay, the expression of SGLT1 in the kidney in the HF + Apoe^−/−^ and HF + Apoe^−/−^+CS groups was upregulated compared with the CON group (*P* < 0.01) (Figure 6A). Compared with the CON group, the expression level of SGLT2 in the kidney was higher in the CON + CS, HF + Apoe^−/−^, and HF + Apoe^−/−^+CS groups (Figure 6B). In addition, based on the Pearson correlation analysis, the expression of SGLT1 in the kidney was positively related with the proportion of the edema area (*R* = 0.670, *P* < 0.001), broken reticular fiber (*R* = 0.278, *P* = 0.031), and glycogen content (*R* = 0.699, *P* < 0.001). Furthermore, the expression of SGLT2 in the kidney was also positively related with the proportion of the edema area (*R* = 0.790, *P* < 0.001), broken reticular fiber (*R* = 0.481, *P* < 0.001), and glycogen content (*R* = 0.856, *P* < 0.001). There were strongly positive correlations between the expression of SGLT1 and SGLT2 in the kidney (*R* = 0.769, *P* < 0.001). The heatmap manifested positive correlations among edema, glycogen, reticular fiber, and expression of SGLT1 and SGLT2 in the kidney based on the Spearman rho (Figure 6C).

### Sensitivity and specificity of diagnosis of SGLT1 and SGLT2 for edema, reticular fiber, and glycogen content in the kidney

**Figure 7:**
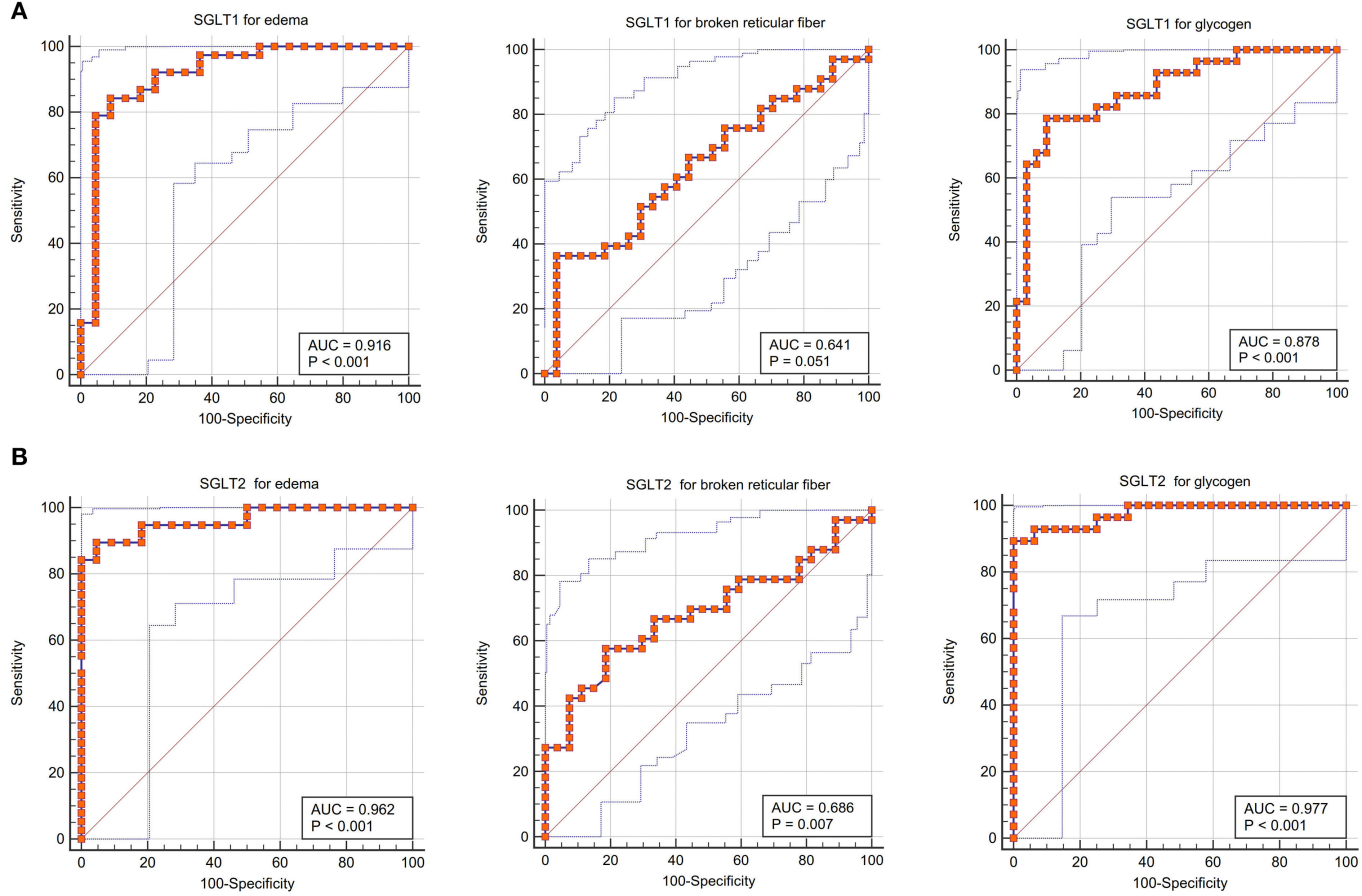
Sensitivity and specificity of diagnosis of SGLT1 and SGLT2 for edema, reticular fibers, and glycogen in the kidney. **(A)** Receiver operating characteristic curve indicated that the expression level of SGLT1 in our experiment also could predict renal edema [area under the curve (AUC) = 0.916; *P* < 0.001], broken reticular fibers (AUC = 0.641, *P* = 0.051), and the glycogen content (AUC = 0.878, *P* < 0.001) sensitively and specifically. **(B)** Expression level of SGLT2 in our experiment also could predict renal edema (AUC = 0.962; *P* < 0.001), broken reticular fibers (AUC = 0.686, *P* = 0.007), and the glycogen content (AUC = 0.977, *P* < 0.001) sensitively and specifically.

The receiver operating characteristic curve indicated that the expression level of SGLT1 in our experiment also could predict renal edema [area under the curve (AUC) = 0.916; *P* < 0.001], broken reticular fibers (AUC = 0.641, *P* = 0.051), and the glycogen content (AUC = 0.878, *P* < 0.001) sensitively and specifically. In addition, the expression level of SGLT2 in our experiment also could predict renal edema (AUC = 0.962; *P* < 0.001), broken reticular fibers (AUC = 0.686, *P* = 0.007), and the glycogen content (AUC = 0.977, *P* < 0.001) sensitively and specifically (Figure 7).

### Predictive value of SGLT1 and SGLT2 for edema, reticular fibers, and the glycogen content in the kidney *via* the support vector machine

**Figure 8:**
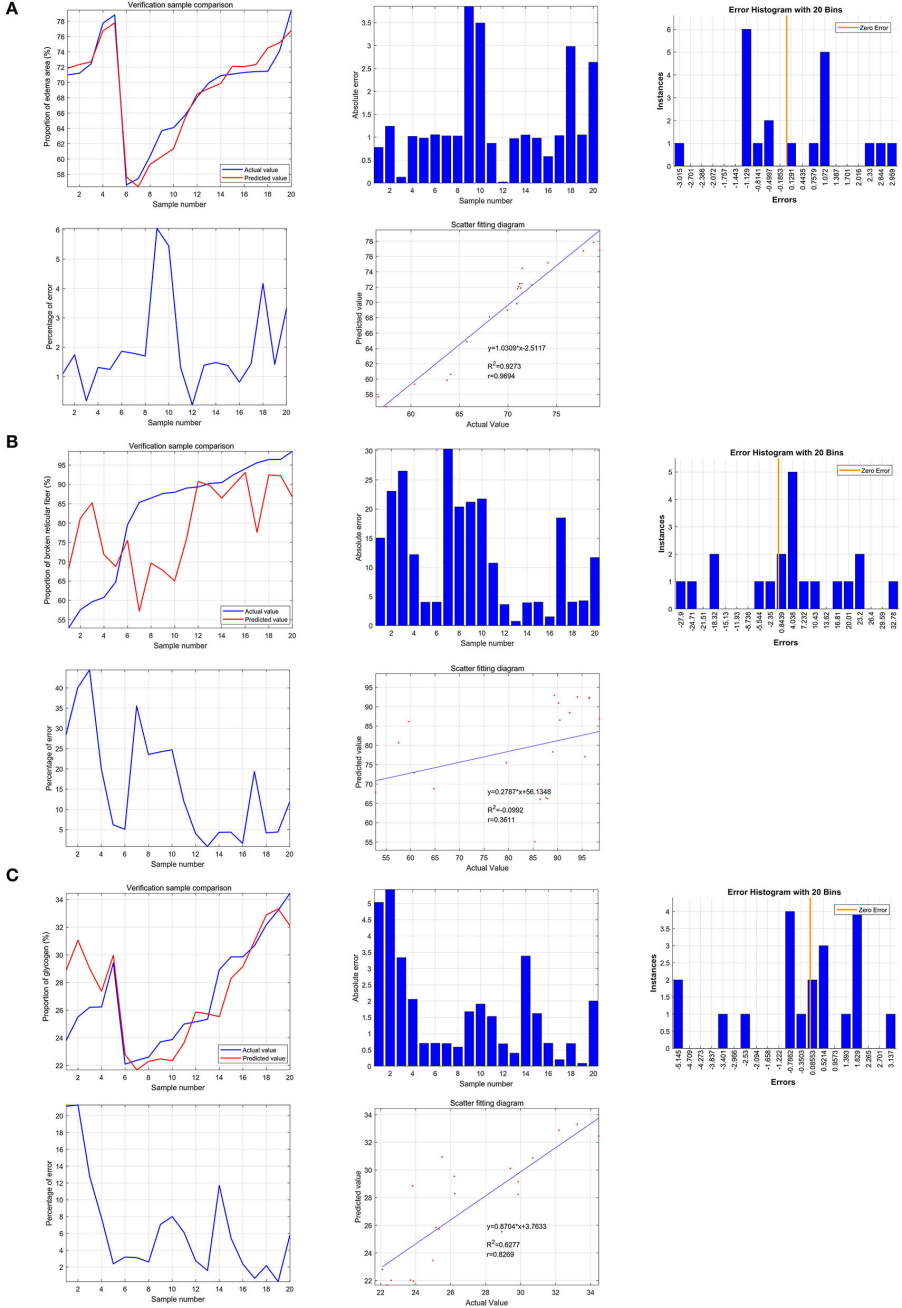
Predictive value of SGLT1 and SGLT2 for edema, reticular fibers, and glycogen content in the kidney *via* the support vector machine (SVM). **(A)** Predictive value of SGLT1 and SGLT2 for the renal edema was 0.9694 (*y* = 1.0309*x−2.5117), and the mean of error was 1.28%. **(B)** Predictive value of SGLT1 and SGLT2 for broken reticular fibers was 0.3611 (*y* = 0.2787*x+56.1348), and the mean of error was 11.62%. **(C)** Predictive value of SGLT1 and SGLT2 for the glycogen content was 0.8269 (*y* = 0.8704*x+3.7633), and the mean of error was 1.56%.

Based on the support vector machine, the predictive value of SGLT1 and SGLT2 for the renal edema was 0.9694 (*y* = 1.0309^*^x−2.5117), and the mean of error was 1.28%. In addition, the predictive value of SGLT1 and SGLT2 for the broken reticular fiber was 0.3611 (*y* = 0.2787^*^x + 56.1348), and the mean of error was 11.62%. Furthermore, the predictive value of SGLT1 and SGLT2 for the glycogen content was 0.8269 (*y* = 0.8704^*^x + 3.7633), and the mean of error was 1.56% (Figure 8).

### The neural network prediction model and high-risk warning range of edema, reticular fibers, and the glycogen content in the kidney

**Figure 9:**
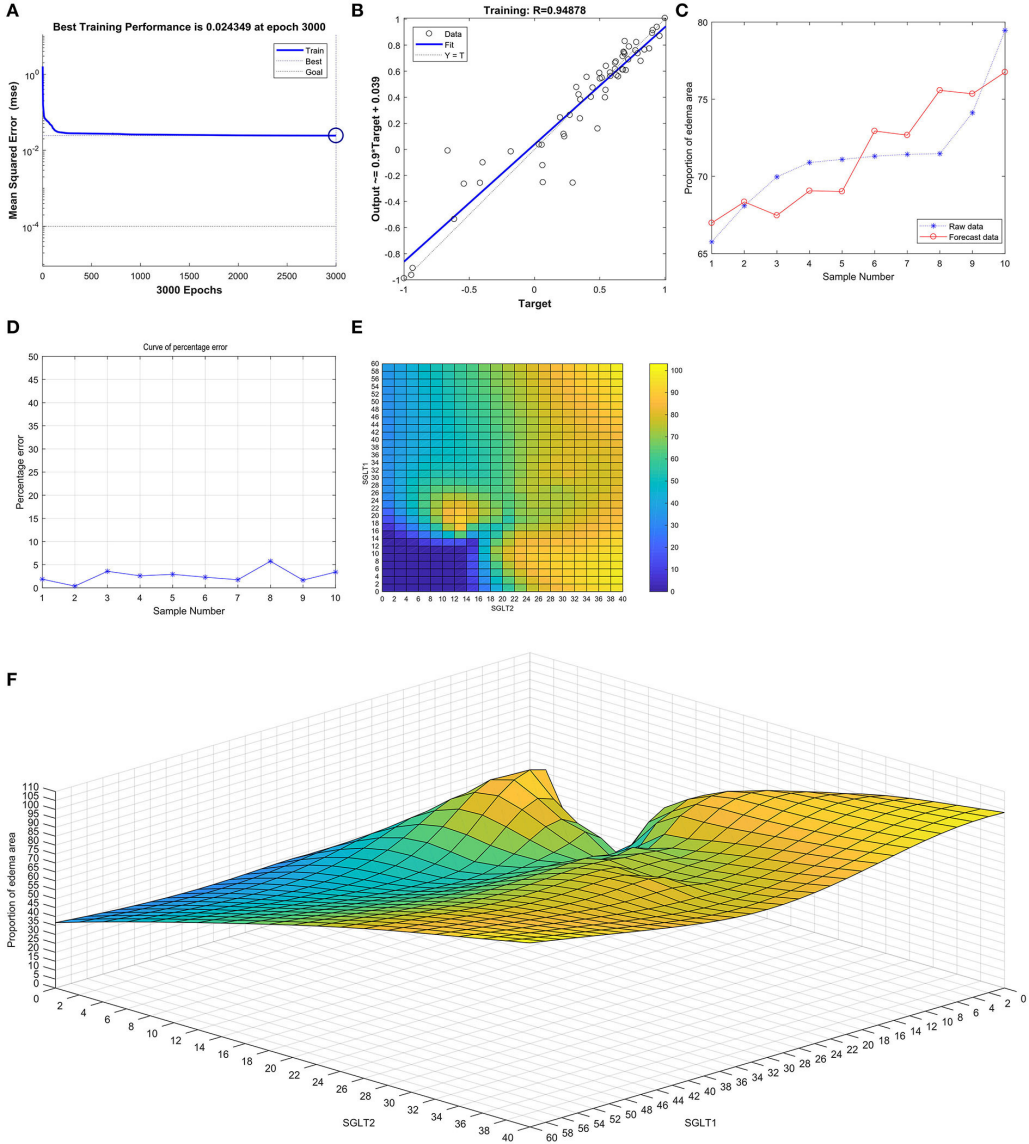
Neural network prediction model and high-risk warning range of renal edema based on the expression of SGLT1 and SGLT2. **(A)** After training the BP neural network of SGLT1 and SGLT2 for renal edema, the best training performance was 0.024349 at epoch 3000. **(B)** Relativity was 0.94878. **(C,D)** By verifying the forecast data against the raw value, we found that there were only small differences. **(E,F)** High-risk warning indicator of renal edema: 16 < SGLT1 < 26 and 8 < SGLT2 < 16.

After training the BP neural network of SGLT1 and SGLT2 for renal edema, the best training performance was 0.024349 at epoch 3,000 (Figure 9A), and the relativity was 0.94878 (Figure 9B). By verifying the forecast data against the raw value, we found that there were only small differences (Figures 9C,D). Based on the obtained result, we could speculate that the expression of SGLT1 and SGLT2 might be the predictive indexes of renal edema. Through the cubic spline interpolation algorithm, we found the high-risk warning indicator of renal edema: 16 < SGLT1 < 26 and 8 < SGLT2 < 16 (Figure 9E). Furthermore, the three-dimensional stereogram could present the warning range well (Figure 9F).

**Figure 10:**
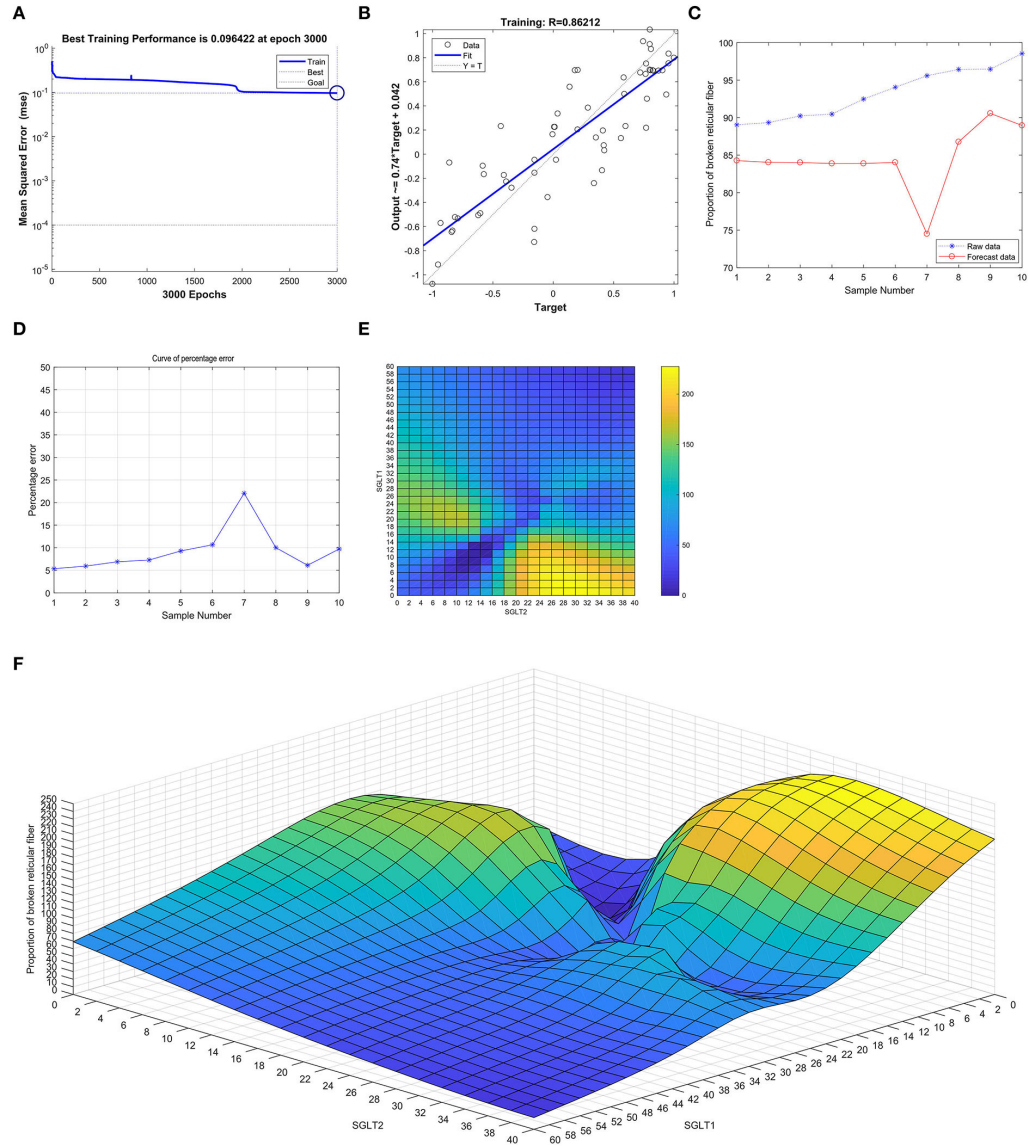
Neural network prediction model and high-risk warning range of broken reticular fibers in the kidney based on the expression of SGLT1 and SGLT2. **(A)** After training the BP neural network of SGLT1 and SGLT2 for broken reticular fibers in the kidney, best training performance was 0.096422 at epoch 3,000. **(B)** Relativity was 0.86212. **(C,D)** By verifying the forecast data against the raw value, we found that there were large differences. **(E,F)** High-risk warning indicator of broken reticular fibers in the kidney: 0 < SGLT1 < 16 and 18 < SGLT2 < 40.

After training the BP neural network of SGLT1 and SGLT2 for broken reticular fibers in the kidney, the best training performance was 0.096422 at epoch 3,000 (Figure 10A) and the relativity was 0.86212 (Figure 10B). By verifying the forecast data against the raw value, we found that there were big differences (Figures 10C,D). Based on the aforementioned result, we could speculate that the expression of SGLT1 and SGLT2 might be the predictive indexes of broken reticular fibers in the kidney. Through the cubic spline interpolation algorithm, we found the high-risk warning indicator of broken reticular fibers in the kidney: 0 < SGLT1 < 16 and 18 < SGLT2 < 40 (Figure 10E). Furthermore, the three-dimensional stereogram could present the warning range well (Figure 10F).

**Figure 11:**
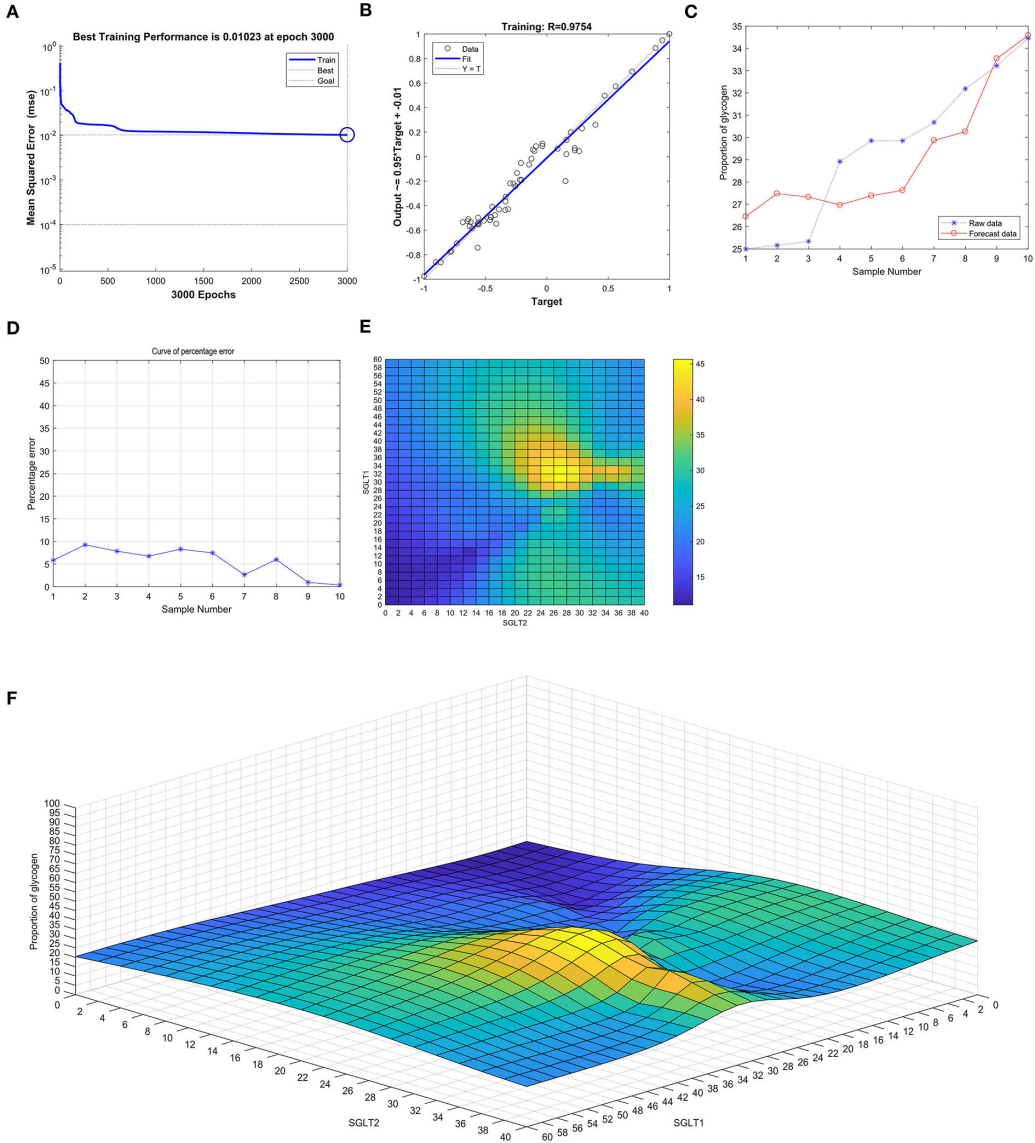
Neural network prediction model and high-risk warning range of the glycogen content in the kidney based on the expression of SGLT1 and SGLT2. **(A)** Best training performance was 0.01023 at epoch 3,000. **(B)** Relativity was 0.9754. **(C,D)** Through verifying the forecast data against the raw value, we found that there were only small differences. **(E,F)** High-risk warning indicator of glycogen content in the kidney: 28 < SGLT1 < 42 and 20 < SGLT2 < 42.

After training the BP neural network of SGLT1 and SGLT2 for the glycogen content in the kidney, the best training performance was 0.01023 at epoch 3,000 (Figure 11A) and the relativity was 0.9754 (Figure 11B). By verifying the forecast data against the raw value, we found that there were only small differences (Figures 11C,D). Based on the aforementioned result, we could speculate that the expression of SGLT1 and SGLT2 might be the predictive indexes of the glycogen content in the kidney. Through the cubic spline interpolation algorithm, we found the high-risk warning indicator of the glycogen content in the kidney: 28 < SGLT1 < 42 and 20 < SGLT2 < 42 (Figure 11E). Furthermore, the three-dimensional stereogram could present the warning range well (Figure 11F).

## Discussion

Atherosclerosis (AS) is the main cause of coronary heart disease, cerebral infarction, and peripheral vascular disease (11). Chronic stress is prevalent in modern society and causes many kinds of mental diseases (12, 13). The main results of this study were that renal SGLT1 expression was increased in the case of chronic stress alone, but not statistically significant (*P* > 0.05). In addition, atherosclerosis alone or atherosclerosis combined with chronic stress significantly promoted renal SGLT1 expression (*P* < 0.05). In the case of chronic stress alone, renal SGLT2 expression increased. In addition, the combination of atherosclerosis and chronic stress will promote renal SGLT2 expression.

SGLTI is mainly distributed in the small intestine and proximal tubules of nephrons. SGLTI plays an important role in the absorption of glucose in the small intestines and is expressed in the brush border of small intestinal epithelial cells (8, 14). In the ratio of 2:1, SGLT1 is a transporter with high affinity and low transport capacity, located in the distal segment of the proximal tubule (S3 segment), and is responsible for 10% glucose reabsorption. In addition, SGLT1 is expressed in intestinal endocrine cells, hepatobiliary duct cells, type II alveolar epithelium, Clara cells, cardiomyocytes, and intracerebral cells. The main results of this study were that renal SGLT1 expression was increased in the case of chronic stress alone, but not statistically significant (*P* > 0.05). In addition, atherosclerosis alone or atherosclerosis combined with chronic stress significantly promoted renal SGLT1 expression (*P* < 0.05). Oxidative damage of vascular endothelial cells is the initial link leading to atherosclerosis AS. Protecting endothelial cells from oxidative damage and maintaining their structure and function are the key to preventing AS (15). Sodium glucose cotransporter I (SGLT1) (16) is an important transporter that mainly mediates the transmembrane transport of glucose and various molecules as SGLT1 role in transportation depends on the presence of Na^+^. Delphinine glucoside (DP) is an anthocyanin compound and can inhibit oxidative stress injury of vascular endothelial cells. Studies have shown that the DP molecule has high antioxidant and ROS scavenging biological activity and can protect endothelial cells from peroxidation damage by scavenging free radicals through antioxidants (17). The decreased expression level of SGLT1 could significantly reduce the absorption rate of endothelial cells to DP. These results suggest that SGLT1 plays an important role in the process of DP uptake in endothelial cells, and the absorption of DP is at least partly dependent on SGLT1. Therefore, when atherosclerosis occurs, the body stimulates the expression of SGLT1 to maintain homeostasis. Therefore, it is speculated that SGLT1 may be a molecular target of chronic stress combined with atherosclerosis in kidney injury.

Compared with SGLTI, SGLT2 is the most important glucose transporter in the kidney. It is a transporter with low affinity and high transport capacity. It is mainly distributed in the S1 and S2 segments of the proximal tubule, is transported at a ratio of sodium to glucose 1:1, and is responsible for more than 90% of glucose reabsorption (8, 14, 18). Familial renal glycosuria (FRG) is caused by mutations in the SGLT2 gene that lead to a normal or below normal renal glycosuria threshold (19, 20). In the case of chronic stress alone, renal SGLT2 expression increased. In addition, atherosclerosis combined with chronic stress will significantly promote renal SGLT2 expression. Elevated blood glucose can increase the formation of advanced glycosylation end products and increase the production of reactive oxygen species (ROS) through signal transduction of specific receptors, leading to vascular dysfunction and damage to target organs. Since endothelial cells hardly express SGLT2, canagliflozin may indirectly activate the Akt-eNOS pathway through its hypoglycemic effect and reduce the inflammatory response (21, 22). Endothelial dysfunction is the initiating link of AS in mice, and SGLT2 inhibitors prevent the occurrence and development of AS by protecting the endothelial function. Existing research data show that SGLT2 inhibitors have a significant effect on a variety of risk factors such AS hyperglycemia, hypertension, abnormal lipid metabolism, and obesity, as well as the functions of endothelial cells, macrophages, and smooth muscle cells, which are closely related to the occurrence and development of AS (23–25). However, SGLT2 is mainly found in the kidney and hardly expressed in the cardiovascular system and other tissues and organs. It is possible that SGLT2 inhibitors exert anti-AS effects through SGLT1 or similar transporter bodies. Therefore, it is speculated that SGLT2 may be a molecular target of chronic stress combined with atherosclerosis on kidney injury.

Many studies have used SGLT2 as a molecular target to specifically inhibit SGLT2, which has become a new approach to insulin-independent treatment of diabetes. Subsequent studies found that SGLT1 plays an important role in glucose transport during normal renal glucose reabsorption. However, after the use of SGLT2 inhibitors, the ability of SGLT1 to transport glucose was enhanced. The heterozygous mice did not suffer severe gastrointestinal adverse reactions (26), suggesting that partial inhibition of SGLT1 can be tolerated by the body. Compared with only inhibition of SGLT2, if the inhibitor can simultaneously inhibit 30% of SGLT1, urine glucose can be increased by 80% (27). SGLT1 is also a major transporter for intestinal glucose absorption; similar to the renal overexpression of SGLT2, SGLT1 is also overexpressed in the gastrointestinal tract of T2DM patients, and inhibition of SGLT1 can also reduce intestinal glucose absorption. Therefore, the inhibition of SGLT2 and partial inhibition of SGLT1 can increase urinary glucose excrement, inhibit gastrointestinal glucose absorption and better control of blood glucose without increasing serious adverse reactions, thus becoming a new hope for the treatment of T2DM.

Although rigorous animal experimental analysis is carried out in this study, there are still few limitations. In this study, no human samples were used to further verify their function. Second, the interaction between SGLT1 and SGLT2 was not studied in the research. Therefore, in aspects of future direction, the researchers should obtain human ethical approval and then recruit patients with atherosclerosis to analyze the role of SGLT1/2 on kidney injury under chronic stress. Furthermore, the molecular docking study should be performed to study the interaction between SGLT1 and SGLT2. In future, studies should carry out in-depth exploration in this aspect.

In conclusion, atherosclerosis alone or atherosclerosis combined with chronic stress can significantly promote renal SGLT1 expression. In the case of chronic stress alone, renal SGLT2 expression was upregulated. In addition, the combination of atherosclerosis and chronic stress will promote renal SGLT2 expression.

## Data availability statement

The original contributions presented in the study are included in the article/supplementary material, further inquiries can be directed to the corresponding author/s.

## Ethics statement

The animal study was reviewed and approved by Animal Care and Use Committee of the Institute of Laboratory Animal Sciences, Chinese Academy of Medical Sciences (CAMS) and Peking Union Medical College (No. LDP21001).

## Author contributions

TG, D-pL, ZC, and Y-jL: conceptualization. L-bM, J-yL, and M-jS: formal analysis. D-pL: funding acquisition and writing—review and editing. M-jS and H-xX: investigation. D-sW: methodology. Y-hC: software. G-fH, XW, and L-bM: supervision and writing—original draft. J-pX: visualization. All authors contributed to the article and approved the submitted version.

## Funding

This present study was funded by the National Key R&D Program of China (Grant Nos. 2020YFC2003000 and 2020YFC2003001), Central Health Research Project (W2017BJ11), and National High Level Hospital Clinical Research Funding (No. BJ-2022-141).

## Conflict of interest

The authors declare that the research was conducted in the absence of any commercial or financial relationships that could be construed as a potential conflict of interest.

## Publisher's note

All claims expressed in this article are solely those of the authors and do not necessarily represent those of their affiliated organizations, or those of the publisher, the editors and the reviewers. Any product that may be evaluated in this article, or claim that may be made by its manufacturer, is not guaranteed or endorsed by the publisher.

## Abbreviations

CS: chronic stress
HF: high fat
HE: hematoxylin–eosin
SVM: support vector machine
ROC: receiver operating characteristic
CON: control
SNS: sympathetic nervous system
HPA: hypothalamic–pituitary–adrenal axis
SGLTs: sodium–glucose cotransporters
RMSE: root mean square error
MAE: mean absolute error
MBE: mean deviation
BP: back propagation
AS: atherosclerosis
DP: delphinine glucoside
FRG: familial renal glycosuria
ROS: reactive oxygen species.
